# O-GlcNAcylation: a pro-survival response to acute stress in the cardiovascular and central nervous systems

**DOI:** 10.1186/s40001-024-01773-z

**Published:** 2024-03-16

**Authors:** Qiu Xue, Shengtao Ji, Hui Xu, Shu Yu

**Affiliations:** 1 Key Laboratory of Neuroregeneration of Jiangsu and Ministry of Education, Co-Innovation Center of Neuroregeneration, NMPA Key Laboratory for Research and Evaluation of Tissue Engineering Technology Products, Nantong University, 19 Qixiu Road, Nantong, 226001 China; 2 Department of General Surgery, Nantong Tumor Hospital, Nantong Fifth People’s Hospital, Affiliated Tumor Hospital of Nantong University, 30 Tongyang North Road, Nantong, 226361 China; 3 Department of Neurology, Affiliated Hospital of Nantong University, Medical School of Nantong University, 20 Xisi Road, Nantong, 226001 China; 4 Nantong Institute of Genetics and Reproductive Medicine, Affiliated Maternity & Child Healthcare Hospital of Nantong University, 399 Century Avenue, Nantong, 226001 China

**Keywords:** O-GlcNAcylation, Stress tolerance, Cardioprotection, Neuroprotection, Hexosamine biosynthetic pathway

## Abstract

O-GlcNAcylation is a unique monosaccharide modification that is ubiquitously present in numerous nucleoplasmic and mitochondrial proteins. The hexosamine biosynthesis pathway (HBP), which is a key branch of glycolysis, provides the unique sugar donor UDP-GlcNAc for the O-GlcNAc modification. Thus, HBP/O-GlcNAcylation can act as a nutrient sensor to perceive changes in nutrient levels and trigger O-GlcNAc modifications of functional proteins in cellular (patho-)physiology, thereby regulating diverse metabolic processes. An imbalance in O-GlcNAcylation has been shown to be a pathogenic contributor to dysfunction in metabolic diseases, including type 2 diabetes, cancer, and neurodegeneration. However, under acute stress conditions, protein O-GlcNAc modification exhibits rapid and transient upregulation, which is strongly correlated with stress tolerance and cell survival. In this context, we discuss the metabolic, pharmacological and genetic modulation of HBP/O-GlcNAc modification in the biological system, the beneficial role of O-GlcNAcylation in regulating stress tolerance for cardioprotection, and neuroprotection, which is a novel and rapidly growing field. Current evidence suggests that transient activation of the O-GlcNAc modification represents a potent pro-survival signalling pathway and may provide a promising strategy for stress-related disorder therapy.

## Introduction

Glycosylation is a posttranslational modification (PTM) characterized by the covalent attachment of glycans to proteins, that occurs in 50%-70% of human proteins [1]. Unlike classic protein glycosylation (*N*-glycosylation), which occurs mostly via an endoplasmic reticulum–Golgi-dependent secretory pathway in the cell, O-linked *N*-acetylglucosaminylation (O-GlcNAcylation) is a unique PTM that is widely present in the nucleoplasm and mitochondria. O-GlcNAcylation is a highly dynamic signalling modification involving the attachment/removal of *N*-acetylglucosamine (GlcNAc) via an O-linkage with specific serine and threonine residues on proteins, and its function is similar to that of quintessential protein phosphorylation. Since it was first identified on mouse lymphocytes in 1984 [2], O-GlcNAcylation has been shown to regulate a multitude of cellular (patho)physiologies, including type 2 diabetes, cancer, and neurodegeneration [3]. For example, when cells are exposed to chronic hyperglycaemia, high O-GlcNAc levels reduce the effectiveness of insulin signalling pathways via metabolic regulation at the transcriptional level, leading to insulin resistance and type 2 diabetes [4, 5]. However, an increase in O-GlcNAc levels is an endogenous defence response to stress and initially acts in a protective manner. The beneficial effects of an acute and transient increase in O-GlcNAcylation in mediating stress tolerance and cell survival have recently been recognized. In this review, we discuss the beneficial role and potential mechanisms by which O-GlcNAcylation promotes self-tolerance and maintains cellular homeostasis under stress conditions, with a focus on the cardiovascular and central nervous systems (CNS).

## Metabolic, pharmacological, and genetic modulation of O-GlcNAcylation

**Fig. 1:**
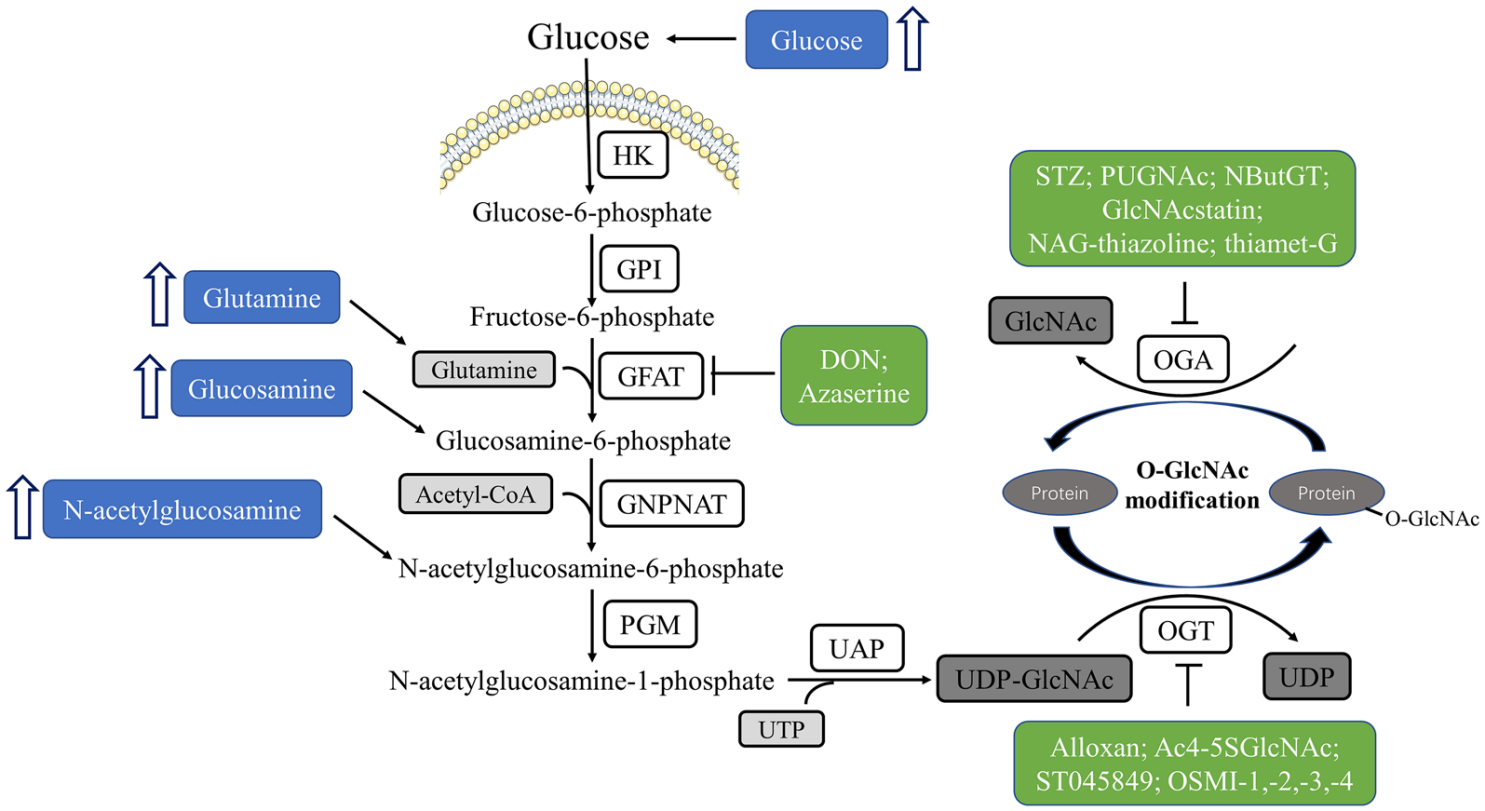
A schematic overview of the hexosamine biosynthesis pathway (HBP) and O-GlcNAcylation. Glucose imported into the cells is rapidly converted to fructose-6-phosphate via the beginning stages of glycolysis. Then, under the catalysis of the rate-limiting enzyme GFAT and other enzymes, HBP integrates multiple metabolic nutrients, ultimately generating UDP-GlcNAc, which is a unique monosaccharide donor for O-GlcNAcylation. The O-GlcNAc cycling is a highly dynamic and reversible modification controlled by a pair of antagonistic enzymes, OGT and OGA. The metabolic and pharmacological interventions for studying the functional role of HBP/O-GlcNAcylation are illustrated with blue and green boxes, respectively

Unlike kinases and phosphatases with substrate specificities, the recycling of O-GlcNAc on proteins is controlled by only one pair of antagonistic enzymes, O-GlcNAc transferase (OGT) and O-GlcNAcase (OGA). This posttranslational modification requires UDP-GlcNAc as its sugar donor, which is synthesized via the hexosamine biosynthesis pathway (HBP), which is a branch of glucose metabolism [6]. The HBP branches off from the beginning stages of glycolysis and ultimately generates UDP-GlcNAc under multistep enzymatic catalysis with the involvement of amino acids (glutamine), fatty acids (acetyl-CoA), and nucleotides (UTP) (Fig. 1). Because multiple metabolites enter the HBP, the levels of UDP-GlcNAc and O-GlcNAc cycling are sensitive to fluctuations in these nutrient intermediates. For example, an increase in HBP flux driven by acute or chronic hyperglycaemia can lead to an increase in UDP-GlcNAc levels, causing the activation of O-GlcNAcylation in multiple cell types [7, 8]. Glutamine is also a potential activator of the HBP. Numerous studies have demonstrated that glutamine enhances stress tolerance and cell survival via HBP flux and increased protein O-GlcNAc levels in the heart and brain [9, 10]. The addition of glucosamine should be an effective means of driving the HBP/O-GlcNAc, since glucosamine can be directly phosphorylated to form glucosamine-6-phosphate by hexokinase, bypassing glutamine-fructose-6-phosphate amidotransferase (GFAT), a key rate-limiting enzyme for the formation of UDP-GlcNAc [11]. Thus, glucosamine is widely and extensively used in the biological systems as a metabolic intervention for functional studies of O-GlcNAcylation. Notably, unlike glucose, high concentrations of glucosamine can overwhelm the biosynthetic capacity of the HBP, causing massive accumulation of glucosamine-6-phosphate, ultimately leading to cellular ATP depletion via allosteric changes in various enzymes. Therefore, the judicious use of glucosamine rather than excessive concentrations of glucosamine facilitates our understanding of insulin resistance induced by hexosamine [12–14]. In addition, GlcNAc also significantly contributes to UDP-GlcNAc biosynthesis and serves as an available means for the increase in O-GlcNAc levels [15, 16]. Therefore, O-GlcNAcylation is highly sensitive to metabolite pools via the HBP and responds quickly to metabolic cues (Fig. 1), representing an important posttranslational mechanism for maintaining cellular homeostasis.

In addition to the aforementioned metabolic interventions, the modulation of O-GlcNAc cycling can be achieved by pharmacological manipulation targeting key regulatory enzymes involved in the HBP/O-GlcNAcylation pathway. GFAT, which is the rate-limiting enzyme in the HBP, is key to controlling HBP flux. Since flux through GFAT is glutamine dependent, HBP flux can be inhibited by glutamine analogues, such as 6-diazo-5-oxo-norleucine (DON) or O-diazoacetyl-l-serine (azaserine). Of note, these substrate analogues have too many off-target effects and potential cytotoxicity [17, 18], therefore, new potent and reversible GFAT inhibitors are being developed [19, 20].

In addition to GFAT, the expression or activity control of OGT and OGA are also targets for intervention. OGT has a high affinity for UDP-GlcNAc, and its affinity for peptides is exquisitely modulated by UDP-GlcNAc levels. In fact, several OGT inhibitors are being widely used to pharmacologically modulate O-GlcNAcylation in functional analyses, including alloxan, a UDP-GlcNAc analogue (Ac4-5SGlcNAc), ST045849, and OSMI-1, − 2, − 3, and − 4. Notably, alloxan is now rarely used as an OGT inhibitor due to its dual inhibitory effects on both OGT and OGA [21]. Moreover, there are several pharmacological inhibitors of OGA, including streptozotocin (STZ), PUGNAc, NButGT, GlcNAcstatin, NAG-thiazoline, and thiamet-G (Fig. 1). Despite the widespread use of these OGT and OGA inhibitors, researchers are still concerned about their potential off-target effects. Thus, the use of genetic approaches to manipulate the level of O-GlcNAc modification, such as RNA interference, adenoviral overexpression or transgenic mouse models, are expected to contribute to the understanding of drug targets and off-target-associated safety. The commonly used and emerging metabolic, pharmacological and genetic interventions for functional studies on HBP/O-GlcNAcylation are listed in Table 1.

**Table 1:** List of commonly used and emerging metabolic, pharmacological, and genetic interventions for studying the functional role of HBP/O-GlcNAcylation in biological systems

Group	Targets	Metabolites/inhibitors/genetic techniques	Effect of O-GlcNAcylation	References
Metabolic interventions	HBP	Glucose	Increase	[7, 8]
		Glutamine	Increase	[9, 10]
		Glucosamine	Increase	[22, 23]
		*N*-acetylglucosamine	Increase	[15, 16]
Pharmacological interventions	GFAT	DON	Decrease	[24, 25]
		Azaserine	Decrease	[9, 26]
	OGT	Alloxan	Decrease	[21, 27]
		Ac_4_-5SGlcNAc	Decrease	[28, 29]
		ST045849	Decrease	[30, 31]
		OSMI-1, − 2, − 3, and − 4	Decrease	[32, 33]
	OGA	STZ	Increase	[34, 35]
		PUGNAc	Increase	[36, 37]
		NButGT	Increase	[38, 39]
		GlcNAcstatin	Increase	[40, 41]
		NAG-thiazoline	Increase	[42, 43]
		thiamet G	Increase	[44, 45]
Genetic interventions	OGT	siRNA	Decrease	[46, 47]
		Adenoviral overexpression	Increase	[46, 47]
		Transgenic mouse models	Increase	[48]
	OGA	siRNA	Increase	[49, 50]
		Adenoviral overexpression	Decrease	[47, 51]
		Transgenic mouse models	Decrease	[48]

## O-GlcNAcylation and stress tolerance

It is well known that organisms have evolved specific stress adaptation strategies to response to environmental fluctuations [52]. Although chronic hyperglycaemia is a potential risk factor for the severity of multiple diseased organs, early and rapid hyperglycaemia caused by stress has been considered an evolutionarily preserved adaptive response that provides a protective effect and supports survival during acute illnesses [53]. Under acute stress, the neuroendocrine response is characterized by activation of the sympathetic nervous system and the massive release of catecholamines, leading to increased secretion of glucagon. Glucagon promotes excessive gluconeogenesis and glycogenolysis, causing stress-induced hyperglycaemia and providing energy for high-energy organs, such as the brain and heart [54]. Along with providing a ready source of fuel, the hypermetabolic state can inhibit glycolytic flux via reactive oxygen species (ROS), thereby increasing the availability of glucose for the HBP/O-GlcNAc pathway [55]. Thus, O-GlcNAc modification is a nutrient and stress sensor, indicating a potential mechanism linking stress-induced hyperglycaemia with beneficial outcomes.

In the past two decades, the beneficial effects of acute stimulation of protein O-GlcNAc levels in the context of stress tolerance and cell survival have received widespread attention. In 2004, Zachara et al. first proposed that O-GlcNAcylation was a stress signalling through which cells rapidly detected and responded to a diverse array of stress stimuli to survive [56]. In fact, numerous reports have demonstrated that transient activation of O-GlcNAcylation is an endogenous adaptation against stress, and metabolic, pharmacological and genetic augmentations of O-GlcNAc levels promote cellular survival in multiple tissues and organs. Next, we focus our discussion on the beneficial role of O-GlcNAcylation in mediating stress tolerance in the cardiovascular system, as well as neuroprotection, which is a novel and rapidly growing field.

## O-GlcNAcylation and cardioprotection

### GIK therapy and O-GlcNAcylation

Glucose–insulin–potassium (GIK) therapy has played a beneficial role in acute myocardial infarction and cardiac surgery over the last 50 years [57, 58]. Although the mechanism by which GIK therapy confers cardioprotection is not known, it is widely accepted that increases in glucose uptake and metabolism are common features of this metabolism-based therapy [59]. Other researchers have suggested that the beneficial effects of GIK therapy can be attributed to an increase in O-GlcNAc signalling. In patients undergoing aortic valve replacement surgery, the improved outcome of low cardiac output after GIK therapy is associated with increased AMPK/Akt phosphorylation and O-GlcNAcylation of selected protein bands [60]. Furthermore, in cultured cardiomyocytes exposed to ischaemic shock, the cytoprotective effect of GIK therapy may involve the inhibition of ROS and upregulation of O-GlcNAcylation and OGT expression [61]. In fact, O-GlcNAcylation can act as a signalling molecule to rapidly respond to nutrient status and play a fundamental role in the endogenous defence of cardiomyocyte survival. For example, a series of studies on isolated perfused rat hearts provided early evidence of the functional relevance of HBP/O-GlcNAc flux and cellular stress tolerance, signifying that acute O-GlcNAc activation was an important PTM that regulated stress survival [9, 34, 62]. Subsequently, many studies have investigated changes in O-GlcNAc modification under stress conditions in various in vitro and in vivo models and explored the functional role of O-GlcNAcylation in mediating myocardial stress tolerance via metabolic, pharmacological and genetic interventions (Table 2). Next, we discuss the functional relevance of O-GlcNAc levels and cellular stress resistance, as well as the specific mechanisms through which O-GlcNAc exerts cardioprotection.

**Table 2:** List of models for myocardial stress tolerance modulated by O-GlcNAc modification

In vitro/ex vivo/in vivo	Models	Cells/tissues/organs/animals	O-GlcNAc levels in stress	O-GlcNAc protection	Methods of modulation	References
In vitro	Heat shock	NRVMs	Increase	Increase	N/A	[63]
	Hypoxia and H/R	NRCMs, NMCMs, CSCs	Increase	Increase	P, G	[46, 50, 51, 64]
	CoCl_2_	HUVECs	N/A	N/A	G	[65]
	I/R	NRVMs	Increase	Increase	M, P, G	[61, 66–69]
	H_2_O_2_	NRCMs	Complex	Increase	P, G	[51, 66, 70]
	ER stress (BfA, TM)	NRCMs	Increase	Increase	P, G	[71]
	LPS	NRVMs, macrophages	N/A	N/A	M, P, G	[23]
	TNF-α	Aortic rings, HUVECs VSMCs	N/A	Increase	M, P	[72, 73]
Ex vivo	I/R, IPC, rIPC	Isolated hearts	Increase	Increase	M, P	[9, 34, 42, 62, 74–76]
In vivo	I/R, IPC	Mice, Murine	Complex	Increase	P	[51, 68–70, 77]
	Hypoxia/ hypoxic acclimation	Mice	Increase	Increase	P	[65, 77]
	Acute arterial injury	Rats	Complex	Increase	M, P	[72, 78]
	Trauma- haemorrhage	Rats	Decrease	Increase	M, P, G	[23, 36, 37, 79]
	Septic shock	Rats	N/A	Increase	P	[38]

Metabolic (M), pharmacological (P), genetic (G) interventions; N/A, not addressed in this model

## Mechanisms by which O-GlcNAcylation confers myocardial stress tolerance

### Calcium and redox homeostasis

The severity of myocardial I/R injury is intimately tied to the sustained increase in intracellular calcium levels (calcium overload). O-GlcNAc signalling has been shown to regulate Ca^2+^-mediated events in cardiomyocytes. In cultured cardiomyocytes acutely treated with glucosamine, an increase in UDP-GlcNAc and O-GlcNAc levels is coupled to the inhibition of calcium overload induced by angiotensin II. This cardioprotection can be simulated by PUGNAc or eliminated by alloxan, indicating a close link between HBP/O-GlcNAc levels and intracellular calcium homeostasis [80]. Subsequently, research on the calcium paradox model of isolated hearts found that short-term high glucose or glucosamine challenge significantly improved cardiac function recovery, while pharmacological inhibition of GFAT or OGT restored sensitivity to the calcium paradox [34]. These cardioprotective mechanisms can be attributed at least in part to the reduction in calcium/calpain-dependent proteolysis, including alpha-fodrin, Ca^2+^/calmodulin (CaM)-dependent protein kinase (CaMKII) [62], and calcineurin [67]. It is worth noting that in a comparative study of K^+^ channel remodelling in hearts exposed to acute and chronic hyperglycaemia, O-GlcNAcylation of CaMKII at Ser-280 enhanced the recovery of K^+^ channels from inactivation during acute hyperglycaemia. However, chronic hyperglycaemia and sustained activation of CaMKII lead to significant arrhythmogenic electrophysiological remodelling [81]. Furthermore, excessive O-GlcNAc modification of CaMKII has been shown to contribute to the induction of ROS, which may exacerbate the pathological consequences of hyperglycaemia in diabetes [7, 82]. Notably, in a recent study on the diabetic heart, the O-GlcNAc modification of the histone deacetylase 4 subdomain at Ser-642, which is an important epigenetic regulator, exerted cardioprotective effects by counteracting pathological CaMKII signalling [83].

Calcium transport pathways are highly sensitive to oxidative stress. Accumulating evidence indicates that ROS and Ca^2+^ signalling likely play central roles in the pathogenesis of cardiovascular dysfunction [84]. Recent work suggests that a dynamic mitochondrial O-GlcNAcylation system rapidly modulates oxidative phosphorylation and ROS release in the heart [39]. In mice exposed to hypoxic acclimation, O-GlcNAc modification of glucose-6-phosphate dehydrogenase increases the NADPH/NADP^+^ and GSH/GSSG ratios, contributing to redox homeostasis in the I/R-exposed heart [77]. O-GlcNAc signalling also attenuates hypoxic/H_2_O_2_-induced Ca^2+^ overload in cultured neonatal rat cardiomyocytes [51]. In addition, a similar protective mechanism of O-GlcNAcylation has been found in the neuronal defence against Aβ neurotoxicity [85]. Interestingly, in human corneal endothelial cells exposed to tBHP (an oxidative stress inducer), the increase in O-GlcNAc signalling induced by PUGNAc reduces intracellular ROS and restores cellular viability, and this beneficial effect is due to the maintenance of mitochondrial calcium homeostasis, indicating that mitochondrial calcium signalling may be a key target for O-GlcNAcylation [86]. Paradoxically, high glucose or thiamet-G treatment promotes the excessive ROS generation in cardiomyocytes via CaMKII O-GlcNAcylation-dependent sarcoplasmic reticulum Ca^2+^ release [7]. The integration of O-GlcNAc into calcium and redox signalling is under intense investigation.

### Mitochondrial homeostasis

In 2008, Ngoh et al. first provided evidence that O-GlcNAcylation plays a fundamental role in mitochondrial homeostasis to influence cardiomyocyte survival/death. They reported that an acute increase in OGT exerts a cardioprotective effect by maintaining the mitochondrial permeability transition pore (mPTP) and mitochondrial membrane potential in the myocardium exposed to hypoxia–reoxygenation insult [46]. Further studies investigating the effects of high glucose or hyperglycaemia in diabetes on myocardial function showed that a chronic increase in O-GlcNAcylation causes mitochondrial dysfunction, including the impairment of mitochondrial respiratory complex activity [87], an imbalance in mitochondrial fusion and fission [88], and mitochondrial DNA (mtDNA) damage [89]. Subsequently, Banerjee et al. reported the presence of mitochondrial-specific OGT, OGA, and UDP-GlcNAc transporters and confirmed that the dysregulation of O-GlcNAc cycling within mitochondria contributed to mitochondrial dysfunction associated with diabetic cardiomyopathy [90]. In fact, mitochondria are targets of O-GlcNAc modification [91]. In a mouse model with impaired branched-chain amino acid catabolism, a reduction in HBP/O-GlcNAc levels selectively disrupted the use of mitochondrial pyruvate by inhibiting pyruvate dehydrogenase complex activity, resulting in a significant decrease in glucose oxidation in the heart [92]. In a study of cardiac I/R injury, the beneficial effect of O-GlcNAcylation induced by hypoxic acclimation was partly attributed to mitochondrial preservation, including effects on mitochondrial ultrastructure, mitochondrial respiration, mtDNA, and mitochondrial redox homeostasis [77]. Redox and calcium handling may be key regulators of mPTP-dependent apoptosis cascade events that occur in mitochondria impaired by stress. The transient opening of the mPTP allows for the release of mitochondrial contents and the activation of intrinsic apoptosis pathways. In cardiomyocytes subjected to hypoxia or oxidative stress, pharmacological and genetic manipulation of OGT and OGA confirmed that O-GlcNAcylation alleviated the formation of mPTP by inhibiting ROS generation and calcium overload [51]. Proteome and O-GlcNAcome analysis of cardiac mitochondria from thiamet-G-treated rats revealed that many mitochondrial proteins, especially those in the oxidative phosphorylation system, are major targets for O-GlcNAcylation. Although certain sites of specific proteins exhibit decreases in O-GlcNAc modification, global protein O-GlcNAc levels are increased, which leads to the enhancement of mitochondrial bioenergetics and the threshold for mPTP opening in the presence of calcium [93].

**Fig. 2:**
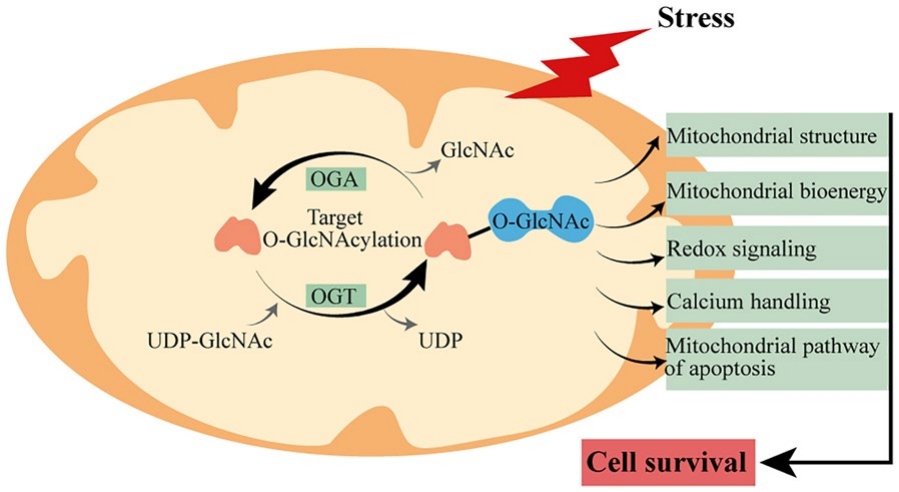
Schematic representation of key targets for O-GlcNAc cycling on mitochondrial homeostasis, including mitochondrial structure, mitochondrial bioenergy, redox signalling, calcium handling, and the mitochondrial apoptosis pathway

Voltage-dependent anion channel (VDAC), which is the primary channel for Ca^2+^ influx and efflux through the outer mitochondrial membrane (OMM) [94], is a target for O-GlcNAcylation. Functional biochemical assessments indicate that the enhanced resistance of mitochondria to mPTP formation induced by calcium is intimately associated with the increase in the number of O-GlcNAc-modified VDACs [70], while inhibiting VDAC O-GlcNAc modification makes mitochondria sensitive to calcium-induced mPTP opening [46]. The functional relevance of VDAC O-GlcNAcylation to mPTP inhibition and cellular tolerance has also been confirmed by the protective effect of the volatile anaesthetic isoflurane on myocardial I/R stress [68]. Although VDAC may not be an essential component of the mPTP, the fact that VDAC participates in mitochondrial membrane permeability and apoptosis signalling by modulating mitochondrial Ca^2+^ flux cannot be ignored [95]. The Bcl-2 family appears to be responsible for the regulation of mitochondrial Ca^2+^ transport systems, including VDAC and the mPTP [96]. The dynamic interactions between Bcl-2 family proteins induce conformational changes in proteins, leading to oligomerization (homologous or heterologous) and membrane insertion, thereby regulating the permeabilization of the OMM and apoptosis [97]. There is evidence that the beneficial effect of O-GlcNAc modification on the maintenance of mitochondrial membrane potential and cytochrome c in stressed cardiomyocytes can be attributed to an increase in mitochondrial Bcl-2 translocation rather than changes in BAD or Bax [66]. However, in H9c2 cardiomyoblasts exposed to chronic hyperglycaemia, excessive O-GlcNAcylation of the proapoptotic protein BAD has been shown to contribute to the formation of the BAD-Bcl-2 dimer, thus enhancing cellular apoptosis [98]. Mitochondrial dysfunction associated with an imbalance in O-GlcNAcylation in the context of glucose toxicity due to hyperglycaemia cannot be ignored. Overall, these studies highlight a profound impact of O-GlcNAcylation on mitochondrial homeostasis, including mitochondrial structure, mitochondrial bioenergy, redox signalling, calcium handling, and the mitochondrial apoptosis pathway (Fig. 2). Further investigation of the multiple layers of complexity between O-GlcNAcylation and mitochondrial homeostasis is needed.

### Endoplasmic reticulum stress

**Fig. 3:**
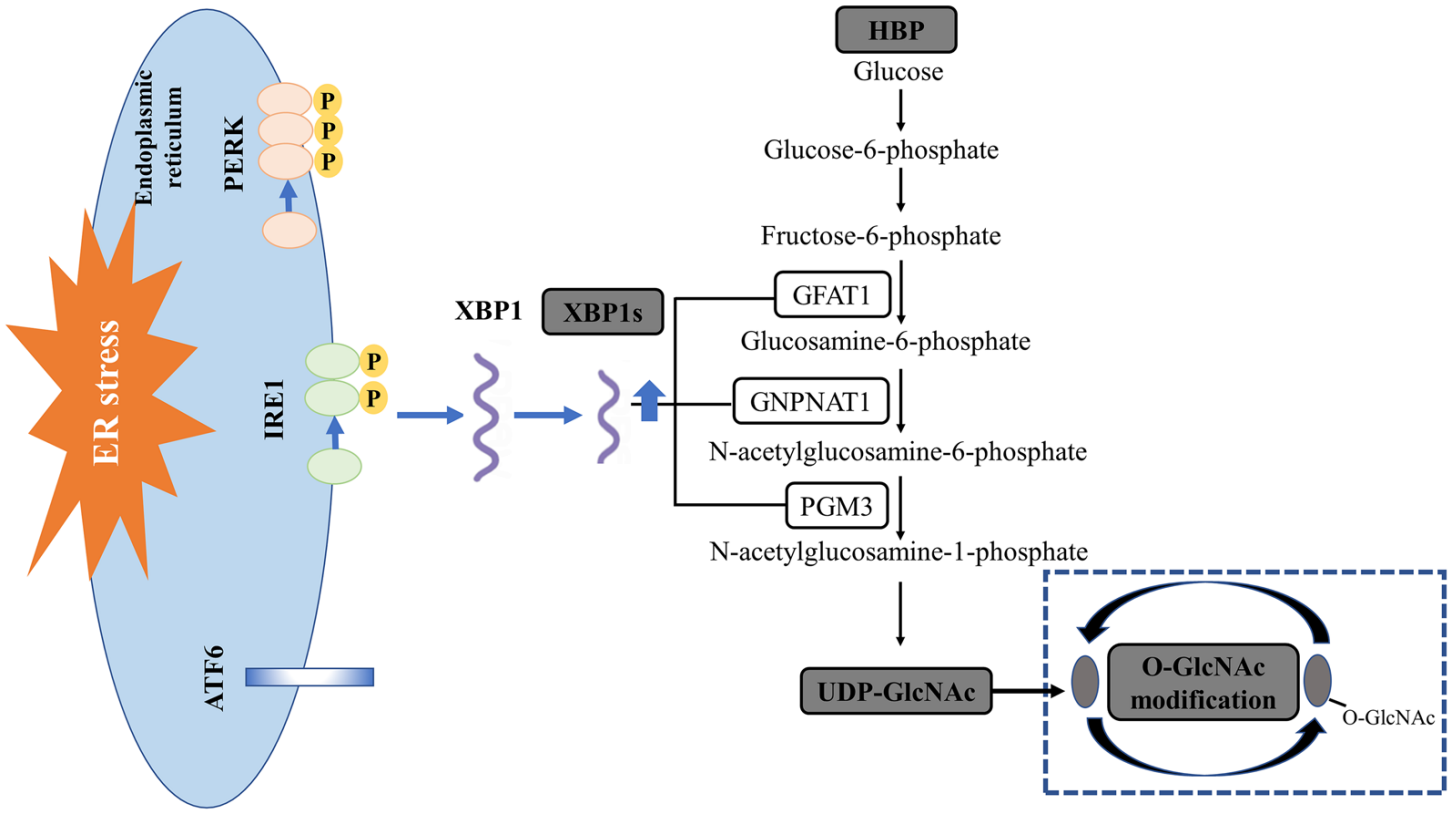
A schematic overview of the XBP1s/HBP/O-GlcNAc axis in the heart and brain

The endoplasmic reticulum (ER) possesses a strict quality control system for protein folding, posttranslational modification, and assembly. The quality control capability of ER is limited. Under pathological conditions, large amounts of unfolded or misfolded proteins accumulate in the ER, resulting in ER stress and the unfolded protein response (UPR). The adaptive UPR plays a beneficial role in restoring protein homeostasis in the ER, while the maladaptive or terminal UPR is involved in the destruction of ER integrity and cellular defects. In metazoans, the UPR includes three signalling pathways: the membrane-anchored transcription factor ATF6, the inositolase IRE1, and the protein kinase PERK [99]. In 2009, Ngoh et al. first proposed that ER stress was a key pathological factor in cardiomyocyte death induced by hypoxia, and increasing O-GlcNAc levels by pharmacological or genetic manipulation mitigated the death of cardiomyocytes exposed to ER stress inducers [71]. In constitutive cardiomyocyte-specific OGT-KO mice, gradual and progressive cardiomyopathy is accompanied by increased expression of ER stress markers, suggesting a close link between ER function and O-GlcNAcylation [100]. Notably, transcriptional activation of the UPR/HBP axis in various stress conditions has been confirmed. The UPR triggers the transcription of key members (GFAT1, GNPNAT1, and PGM3) of the HBP via its most conserved signal transducer spliced X-box binding protein 1 (xbp1s, a transcription factor), leading to the activation of HBP and O-GlcNAcylation, thus providing robust cardioprotection in mice (Fig. 3) [69]. Similar to xbp1s, the ER resident transcription factor spermatogenesis 40 (Tisp40) transcriptionally activates the HBP in conditions of cardiac stress [101]. In turn, O-GlcNAcylation can modulate cellular homeostasis in response to ER stress by modulating eukaryotic translation initiation factor 2α (eIF2α), which is one of the UPR branches [102]. However, this regulatory mechanism has not yet been demonstrated in the cardiovascular system.

### Inflammation

Although it has been reported that the beneficial effects of O-GlcNAc stimulation on improving survival and cardiac function in septic shock are independent of inflammation [38], numerous studies have shown that there is an intimate relationship between inflammation and O-GlcNAcylation in stressed hearts. Glucosamine exerts anti-inflammatory effects on various cell types and models, including systemic inflammation [103] and osteoarthritis [104]. In a rat model of trauma-induced haemorrhage, the increase in O-GlcNAc levels induced by glucosamine or PUGNAc has been shown to improve survival, organ perfusion, and cardiac function. One mechanism of these beneficial outcomes is the attenuation of circulating inflammatory cytokines [36, 37, 79, 105]. Furthermore, the protective effect of the increase in protein O-GlcNAc modification on vascular inflammation and vascular dysfunction has also been confirmed [65, 73, 78]. Importantly, the nuclear factor NF-κB, which is a prototypical proinflammatory signalling factor, is a key molecular bridge linking O-GlcNAcylation and inflammation. For example, an increase in O-GlcNAc levels weakens NF-κB nuclear translocation and subsequent TNF-α and IL-6 expression, thus improving cardiac function following trauma-induced haemorrhage [23]. It is also noteworthy that the NF-κB pathway is involved in vascular inflammation, and glucosamine or thiamet-G treatment has been shown to alleviate inflammation-induced vascular damage by antagonizing the NF-κB signalling cascade [72, 106]. The NF-κB subunit p65 is a target for O-GlcNAcylation, and glycosylation of this factor inhibits self-phosphorylation, thereby preventing p65 downstream signalling [107]. Furthermore, acute O-GlcNAcylation can reduce inducible nitric oxide synthase (iNOS) by inhibiting the NF-κB pathway, thus alleviating oxidative stress-induced vascular dysfunction [73]. Paradoxically, O-GlcNAcylation of NF-κB may also contribute to the lipopolysaccharide (LPS)-induced endothelial inflammatory response [108]. Thus, a deeper investigation of the nuanced relationship between O-GlcNAc and inflammation and how this association impacts cardiac and vascular function under stress conditions is needed.

### Heat shock response

The heat shock response (HSR) is an ancient defence signalling pathway that maintains proteostasis to cope with a variety of cellular stresses. In the HSR, diverse heat shock factors are recruited to control the heat shock protein (HSP)/chaperone network to help modulate protein folding and repair [109]. O-GlcNAc appears to improve the tolerance of cardiomyocytes to multiple forms of stress by upregulating the rates and extent of HSP induction [56, 63, 110, 111], including HSP70, HSP40, HSP72, and aB-crystallin (HSPB5). Many studies associated with protection strategies against myocardial stress have focused on HSP70, a master regulator of protein degradation. Activation of HSP70 enhances the stress adaptation of the myocardium to I/R injury via multiple mechanisms, including oxidative stress, calcium overload, apoptosis, autophagy, and inflammatory responses [112, 113]. Pharmacological enhancement with glutamine induces HSP70 expression and the activation of key transcription factors in the HSP70 pathway in animal models of inflammatory responses [10, 114, 115]. HSP70 exhibits adjustable lectinic activity that depends on glucose concentrations and O-GlcNAc levels [116, 117]. Protein misfolding triggers the release of Hsp70-GlcNAc-binding activity in response to a wide variety of cellular stresses [118]. Importantly, O-GlcNAc signalling prevents proteasome degradation by modifying the specific interactions of HSP70 family members [119]. In addition to HSP70, other HSPs are targets for O-GlcNAcylation, including Hsp90β [120], HSP28 [121], and HSPA6 [122]. However, further studies are needed to determine the role of O-GlcNAcylation in the functional regulation of these HSPs during cellular stress.

Collectively, these results demonstrate that O-GlcNAcylation is a pro-survival signal that mediates myocardial stress tolerance via multiple mechanisms, including calcium and redox homeostasis, mitochondrial homeostasis, ER stress, inflammation, and the HSR.

## O-GlcNAcylation and neuroprotection

Inspired by the beneficial effects of O-GlcNAcylation on improving cardiac function under stress, the discovery and knowledge of the pro-survival response of O-GlcNAcylation in the CNS has exploded recently (Table 3). Extensive work has focused on in vivo cerebral I/R injury experiments in which the activation of O-GlcNAcylation has been shown to be an adaptive response to improve cellular stress tolerance, and increasing this PTM might be a promising strategy for stroke therapy.

**Table 3:** List of models for neuronal stress tolerance modulated by O-GlcNAc modification

In vitro/in vivo	Models	Cells/animals	O-GlcNAc levels in stress	O-GlcNAc protection	Methods of modulation	References
In vitro	OGD/R	Primary neuron cultures, Primary astrocyte cultures, HT22 cells	Increase	Increase	P, G	[123–126]
	LPS	BV2 microglia cells	Increase	Increase	M, P	[44, 127]
	Aβ	Primary cortical neurons , CHO cells	Decrease	Increase	P	[128]
	Glutamate	PC12 cells	Increase	Increase	P, G	[129]
In vivo	Transient global ischaemia, tMCAO, pMCAO	Mice, Rat	Young: increase Aged: no change	Increase	M, P, G	[44, 45, 127, 130–135]
	CA/CPR	Mice	Young: increase Aged: no change	Increase	M, P	[45, 136]
	Hypoxia	Mice	N/A	Increase	P	[137]
	RH	Zebrafish	Decrease	Increase	M	[138]
	SAH	Mice	N/A	Increase	P	[126]

Metabolic (M), pharmacological (P), genetic (G) interventions; N/A, not addressed in this model

### Age-related activation of O-GlcNAcylation

Stroke is an acute cerebrovascular accident that primarily impacts elderly individuals, and clinical evidence shows that the recovery of neurological function worsens with age [139]. Therefore, researchers have focused on comparing cellular responses to ischaemic challenges in young and aged animals in experimental stroke studies and have attempted to determine the role of ageing in the cellular response to severe forms of stress associated with I/R. In a transient forebrain ischaemia model, Liu et al. analysed the activation of proteostasis-related pathways in young and aged mice and found that the most prominent change in the ageing brain was the inactivation of the O-GlcNAc modification, suggesting that this pathway might be a promising target for stroke therapy [132]. In addition to the brain, impaired age-related activation of O-GlcNAcylation has also been confirmed in the kidney and spinal cord after cardiac arrest and cardiopulmonary resuscitation (CA/CPR) [45], signifying the importance of O-GlcNAcylation as a potential mechanism underlying the impairment of functional recovery in ageing organs/tissues in response to ischaemic challenge. LC–MS/MS analysis showed that the availability of UDP-GlcNAc in the aged brain was impaired both at baseline and after I/R, while metabolic intervention with glucosamine significantly improved the acute outcomes in young and elderly mice [130]. Furthermore, other studies have reported that pharmacological increases in O-GlcNAc levels with thiamet-G improved outcomes after ischaemic stroke or CA/CPR in both young and elderly animals [44, 131]. Therefore, interventions targeting the HBP/O-GlcNAc axis might be a promising therapeutic strategy for stroke.

## Mechanisms by which O-GlcNAcylation confers neuronal stress tolerance

### Mitochondrial homeostasis

Mitochondria are crucial for maintaining metabolic homeostasis in the high-energy CNS. In the brain, O-GlcNAc cycling participates in the modulation of mitochondrial network homeostasis, which is diverse and includes mitochondrial trafficking, mitochondrial bioenergetics, mitochondrial fission and fusion, and mitochondrial apoptosis [91]. In fact, under ischaemic stress conditions, O-GlcNAcylation-mediated mitochondrial homeostasis and cellular bioenergetics have emerged as potential pharmacological targets for the development of neuroprotective agents. For example, an active component of *Gastrodia elata* exerts a potent neuroprotective effect by maintaining mitochondrial energy metabolism during cerebral I/R injury. Targeted metabolic profiling suggests that the increased levels of UDP‑GlcNAc and its regulatory enzyme OGT contribute to the beneficial effects of *Gastrodia elata* on stroke [134, 135]. In our laboratory, the compound SalA-4 g was shown to have neuroprotective effects [124]. Specific mechanisms may involve the O-GlcNAc modification of mitochondria by SalA-4 g, which was shown to exert neuroprotective effects by improving mitochondrial homeostasis and inhibiting mitochondrial apoptosis pathways in neurons exposed to ischaemia-like conditions [125]. Recently, in a mouse model exposed to sevoflurane, the beneficial effects of hypoxia acclimation on anaesthetic sensitivity were attributed to the increase in O-GlcNAc-dependent modulation of glutamatergic synapses and mitochondria [137].

Other studies have focused on the functional effects of O-GlcNAc on individual proteins in mitochondria. In neurons, dynamin-related protein 1 (Drp1), which is a critical protein involved in mitochondrial fission, is a target for O-GlcNAcylation [140]. In cerebral I/R injury, the expression of ogt is significantly upregulated, and ogt knockout reduces the phosphorylation of Drp1 Ser-637, leading to the translocation of Drp1 from the cytosol to mitochondria, thus accelerating mitochondria-dependent apoptosis [133]. Another O-GlcNAcylation target, adenosine 5'-triphosphate synthase subunit α (ATP5A), is critically involved in mitochondrial bioenergetics. The decrease in O-GlcNAc modification of the Thr-432 residue on ATP5A induced by Aβ inhibited ATPase activity and disrupted ATP synthesis in Alzheimer's disease (AD) pathology [128]. In neuronal excitotoxicity, the nitric oxide synthase adaptor (NOS1AP) acts as a ligand of neuronal nitric oxide synthases (nNOS) to participate in NMDA receptor-nNOS signalling. Mass spectrometry identified multiple sites for O-GlcNAc modification of NOS1AP, and an increase in this modification prevented its interaction with nNOS, thus protecting against neuronal excitotoxicity induced by glutamate [129].

### XBP1s/HBP/O-GlcNAc axis

The discovery that the UPR branch is involved in the transcriptional activation of HBP/O-GlcNAcylation in cardiac ischaemia has generated a tremendous amount of interest among neuroscientists. In 2017, Jiang and colleagues first reported that the XBP1s/HBP/O-GlcNAc axis was neuroprotective in the context of ischaemic stroke (Fig. 3). They showed that O-GlcNAcylation was activated in an xbp1-dependent manner in the ischaemic penumbra after stroke, and this activation was impaired in the aged brain. Critically, an increase in this response induced by thiamet-G improved short-term stroke outcomes in young and aged mice [131]. Subsequently, further research evaluated and confirmed that thiamet-G improved stroke outcomes in neuron-specific xbp1-knockout mice, including long-term functional recovery. Given the impaired availability of UDP-GlcNAc in the aged brain, the research group further established the beneficial effects of metabolic intervention with glucosamine on stroke models in young and elderly animals [130]. The functional XBP1s/HBP/O-GlcNAc axis, which is a key pro-survival pathway, has also been confirmed in CA/CPR [136] and subarachnoid haemorrhage (SAH) models [126]. Thus, these studies demonstrate that the XBP1s/HBP/O-GlcNAc axis is a promising target for stroke therapy.

### Inflammation

O-GlcNAcylation is involved in controlling inflammatory responses in experimental stroke. Acute increases in O-GlcNAc levels induced by glucosamine [127] or thiamet-G [44] exert neuroprotective effects on the ischaemic brain by inhibiting inflammatory cytokine production and microglial activation. The specific mechanism may involve the inhibition of NF-κB p65 signalling. The similar effects of glucosamine and thiamet-G suggest that suppressing inflammation might contribute to the neuroprotective mechanism of O-GlcNAcylation. Notably, a study on inflammatory modulation in macrophages exposed to LPS suggested that glucosamine could regulate inflammation by sensing different energy states. Under normal and high glucose conditions, glucosamine exerted opposite effects on NO/iNOS production stimulated by LPS depending on energy availability. The bidirectional regulatory effects of glucosamine may contribute to understanding the mechanisms by which O-GlcNAcylation affects nutrient sensing and inflammatory responses [141].

Moreover, dysfunctional O-GlcNAcylation-mediated neuroinflammation has been shown to be involved in the pathology of neurodegeneration. OGT protein levels are significantly low in the cortical neurons of severe AD patients, and specific loss of OGT in the forebrain leads to progressive neurodegeneration, including behavioural and histological phenotypes, as well as extensive gliosis and the upregulation of immune-response genes [142]. In an in vivo zebrafish model of hypoxic brain damage, the downregulation of several glucose metabolites and O-GlcNAc levels may be an important cause of brain inflammation and neurodegeneration, and these changes can be reversed by glucosamine supplementation [138]. In addition to neurons, O-GlcNAcylation is essential for inflammatory responses in astrocytes. The O-GlcNAc modification of NF‑κB p65 has been identified in astrocytes in vitro and in vivo, and increasing O-GlcNAcylation with GlcNAc inhibits inflammation and activation of astrocytes in AD mice by repressing the NF-κB signalling pathway [16]. Collectively, these findings illustrate the beneficial effect of O-GlcNAcylation on stress tolerance by modulating neuroinflammation.

## Conclusions and perspective

The early and rapid hyperglycaemic response to severe injury or trauma is an important adaptive pro-survival process, which is accompanied by an increase in HBP flux and the activation of O-GlcNAc signalling. In animal models and clinical trials, the exact contribution of the HBP/O-GlcNAc pathway to various metabolic-based therapies (high glucose, GIK, and glutamine) has been confirmed. In fact, O-GlcNAc modification can serve as an environmental sensor in metabolic and stress regulation by directly and dynamically modulating protein functions. Numerous studies have demonstrated that the adaptive enhancement of O-GlcNAcylation is a pro-survival signal under stress, and a transient increase in global O-GlcNAc levels induced by stress or interventions (metabolic, pharmacological, or genetic) contributes to stress tolerance, especially in two high-energy organs: the heart and brain. The specific mechanism may involve calcium and redox homeostasis, mitochondrial homeostasis, ER stress, inflammation, and the HSR.

Although the benefits of O-GlcNAcylation in mediating stress tolerance have been clearly recognized, most functional studies still face many challenges. (1) The duration of changes in O-GlcNAc signalling under pathologic conditions (i.e., glucose toxicity and type II diabetes) may have contrary and deleterious effects. The molecular mechanisms underlying the transition from adaptive and pro-survival pathways to pathological responses are still unknown. (2) Due to the potential off-target effects of existing inhibitors, the development of small molecule kinase inhibitors with high specificity and inhibitory effects may contribute to the understanding of drug targets and off-target-associated safety. (3) The tools for identifying the individual O-GlcNAcylation of specific proteins and site-specific O-GlcNAc proteomics (O-GlcNAcomics) are limited. From this perspective, technical advances in high-throughput glycoproteomic studies will provide in-depth insights into the role of O-GlcNAcylation.

## Data Availability

Not applicable.

## Abbreviations

PTM: Posttranslational modification
O-GlcNAcylation: O-Linked *N*-acetylglucosaminylation
GlcNAc: *N*-Acetylglucosamine
CNS: Central nervous system
OGT: O-GlcNAc transferase
OGA: O-GlcNAcase
HBP: Hexosamine biosynthesis pathway
GFAT: Glutamine-fructose-6-phosphate amidotransferase
DON: 6-Diazo-5-oxo-norleucine
azaserine: O-Diazoacetyl-l-serine
STZ: Streptozotocin
HK: Hexokinase
GPI: Glucose-6-phosphate isomerase
GNPNAT: Glucosamine-phosphate *N*-acetyltransferase
PGM: Phosphoglucomutase
UAP: UDP-*N*-acetylglucosamine pyrophosphorylase
ROS: Reactive oxygen species
GIK: Glucose–insulin–potassium
H/R: Hypoxia–reoxygenation
I/R: Ischaemia–reperfusion
BfA: Brefeldin A
TM: Tunicamycin
IPC: Ischaemic preconditioning
rIPC: Remote ischaemic preconditioning
NRVMs: Neonatal rat ventricular myocytes
NRCMs: Neonatal rat cardiac myocytes
NMCMs: Neonatal mouse cardiac myocytes
CSCs: Cardiac stem cells
HUVECs: Human umbilical vein endothelial cells
VSMCs: Vascular smooth muscle cells
CaMKII: Ca^2+^/calmodulin (CaM)-dependent protein kinase
mtDNA: Mitochondrial DNA
mPTP: Mitochondrial permeability transition pore
VDAC: Voltage-dependent anion channel
OMM: Outer mitochondrial membrane
ER: Endoplasmic reticulum
UPR: Unfolded protein response
ATF6: Activating transcription factor 6
IRE1: Inositol-requiring enzyme 1
PERK: Protein kinase RNA-line ER kinase
Xbp1: X-box binding protein 1
Xbp1s: Spliced X-box binding protein 1
Tisp40: Spermatogenesis 40
eIF2α: Eukaryotic translation initiation factor 2α
GNPNAT1: Glucosamine-phosphate *N*-acetyltransferase 1
PGM3: Phosphoglucomutase 3
iNOS: Inducible nitric oxide synthase
LPS: Lipopolysaccharide
HSR: Heat shock response
HSPs: Heat shock proteins
OGD/R: Oxygen–glucose deprivation/reoxygenation
tMCAO: Transient middle cerebral artery occlusion
pMCAO: Permanent middle cerebral artery occlusion
CA/CPR: Cardiac arrest/cardiopulmonary resuscitation
RH: Repetitive hypoxia
SAH: Subarachnoid haemorrhage
HT22 cells: Mouse hippocampal neuronal cells
CHO cells: Chinese hamster ovary cells
PC12 cells: Rat pheochromocytoma cells
Drp1: Dynamin-related protein 1
ATP5A: Adenosine 5'-triphosphate synthase subunit α
AD: Alzheimer's disease
NOS1AP: Nitric oxide synthase adaptor
nNOS: Neuronal nitric oxide synthases
